# Unilateral Sacroiliac Hemiagenesis in a Young Woman Presenting With Chronic Mechanical Low Back and Buttock Pain: A Case Report

**DOI:** 10.7759/cureus.117462

**Published:** 2026-10-05

**Authors:** Manal El Morjani, Ali Rouffa Houmed, Zineb Baba, Ahmed Mougui, Ahlam Belkhou

**Affiliations:** 1 Rheumatology, Arrazi Hospital, Mohammed VI University Hospital Center, Marrakech, MAR; 2 Rheumatology, Faculty of Medicine and Pharmacy of Marrakech, Cadi Ayyad University, Marrakech, MAR

**Keywords:** adulte-onset low back pain, caudal regression syndrome, compensatory scoliosis, sacral agenesis, sacroiliac hemiagenesis

## Abstract

Sacral agenesis is a rare congenital malformation belonging to the spectrum of caudal regression syndrome, whose clinical manifestations are highly variable and depend on the phenotype of the disease.

We report the case of a young woman who presented with chronic mechanical low back and buttock pain. Clinical examination revealed no significant abnormalities. Imaging studies, however, showed right-sided sacroiliac hemiagenesis associated with compensatory scoliosis. After surgical treatment was deemed unnecessary, the patient was managed conservatively with analgesics, muscle relaxants, and an individualized rehabilitation program. Her symptoms gradually improved, with a favorable clinical outcome.

This case represents a mildly symptomatic form of sacral agenesis and emphasizes the important role of imaging in identifying these rare congenital abnormalities, which may otherwise remain unrecognized.

## Introduction

Sacral agenesis is a rare congenital anomaly resulting from abnormal development of the caudal spine. Its clinical presentation is highly variable, ranging from severe forms associated with neurological, urogenital, and musculoskeletal abnormalities to milder forms that may remain unnoticed for years [1,2]. The extent of the anatomical defect largely determines the clinical manifestations, and limited forms may be difficult to recognize when symptoms are mild or nonspecific [2].

Imaging plays an important role in identifying sacral abnormalities and assessing associated spinal and pelvic changes [1,3]. Although sacral agenesis is usually recognized early in life, less extensive forms may occasionally be discovered later during the investigation of persistent musculoskeletal symptoms. We report an unusual case of unilateral sacroiliac hemiagenesis diagnosed in adulthood in a patient presenting with chronic mechanical low back and buttock pain.

## Case presentation

A 36-year-old woman with no significant medical history presented with a 7-year history of mechanical right-sided low back and buttock pain. Over the years, she had received several courses of symptomatic treatment, including different classes of non-steroidal anti-inflammatory drugs (NSAIDs) and step I and II analgesics, administered orally or by injection, with only temporary and incomplete pain relief. The patient was in severe pain, rated at 8/10 on the visual analog scale (VAS), and was able to walk only with assistance.

Examination of the spine in the standing position revealed a scoliotic posture with moderate leftward lumbar deviation. Inspection showed mild flank asymmetry, without significant global trunk imbalance or pelvic tilt. The shoulders were generally level. Clinical examination also revealed a 1.5-cm leg-length discrepancy. The Adam forward bend test demonstrated a moderate left lumbar prominence, with no significant thoracic prominence. Palpation revealed tenderness on percussion of the lumbar spinous processes, with associated paravertebral muscle spasm. Spinal mobility was generally preserved, with mild limitation of left lateral bending. The straight leg raise test (Lasègue’s test) was positive on the right side. Examination of the sacroiliac joints revealed tenderness on direct pressure over the right sacroiliac joint. Both the Patrick (FABER) test and the tripod test were positive on the right side. Mobilization of the right hip was painful, although the range of motion remained preserved. The neurological examination of the lower extremities was normal, with no sensory or motor deficits, no abnormalities in osteotendinous reflexes, and no sphincter dysfunction. Based on these clinical findings, radiological investigations were performed.

A full-spine X-ray revealed a left-curving lumbar scoliosis with a Cobb angle of 24°, associated with a left-curving dorsolumbar scoliosis with a Cobb angle of 6° and a right-curving thoracic scoliosis with a Cobb angle of 5° (Figure 1).

**Figure 1:**
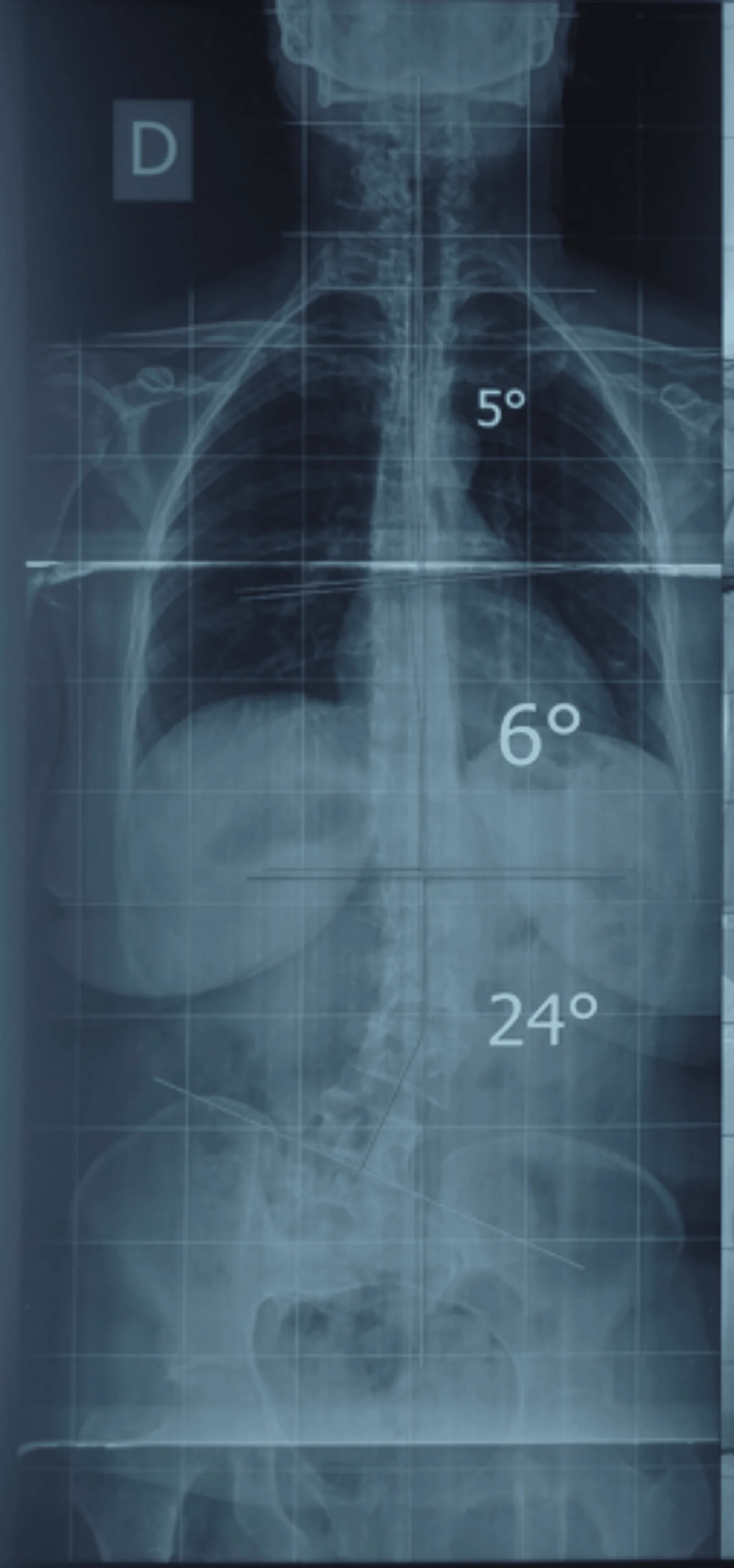
Full-spine X-ray A full-spine X-ray revealed a left-curving lumbar scoliosis with a Cobb angle of 24°, along with a left-curving dorsolumbar scoliosis with a Cobb angle of 6°, and a right-curving thoracic scoliosis with a Cobb angle of 5°.

Magnetic resonance imaging (MRI) of the lumbar spine and sacroiliac joints was performed to better evaluate the disc-vertebral and pelvic structures. This revealed right-sided hemiagenesis - partial agenesis of the sacral wing and complete agenesis of the right sacroiliac joint - which was responsible for pelvic imbalance with compensatory lumbar scoliosis. The MRI also revealed a left L4-L5 foraminal disc herniation coming into contact with the L4 nerve root. A computed tomography (CT) scan was subsequently performed for a more detailed assessment of the bony structures and confirmed the right-sided sacroiliac hemiagenesis (Figure 2 and Figure 3).

**Figure 2:**
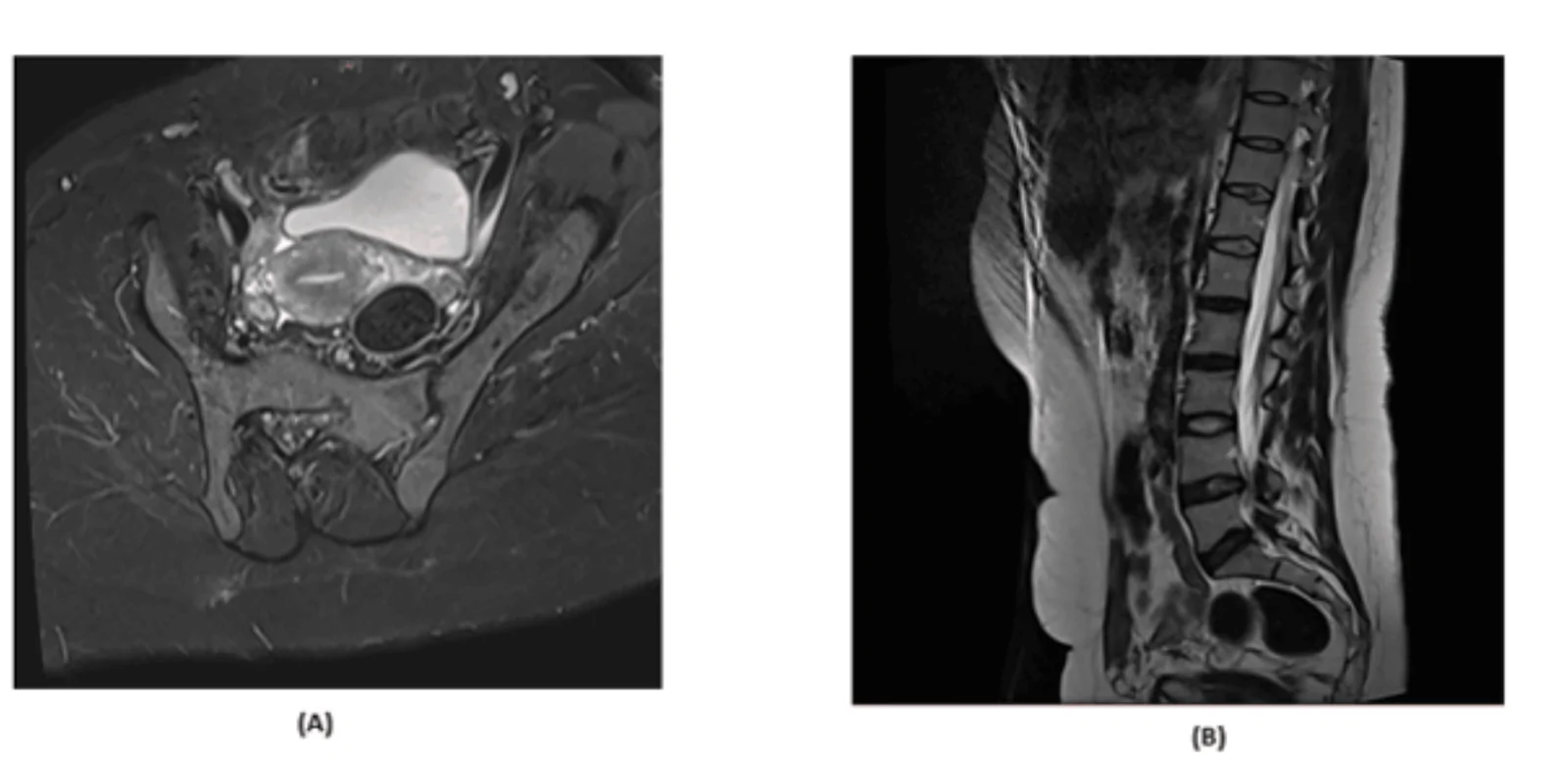
Magnetic resonance imaging of the pelvis and lumbar spine Magnetic resonance imaging of the pelvis (A) and the lumbar spine (B) showing right-sided hemiagenesis - partial agenesis of the sacral wing and complete agenesis of the right sacroiliac joint - and a left L4-L5 foraminal herniation coming into contact with the L4 nerve root.

**Figure 3:**
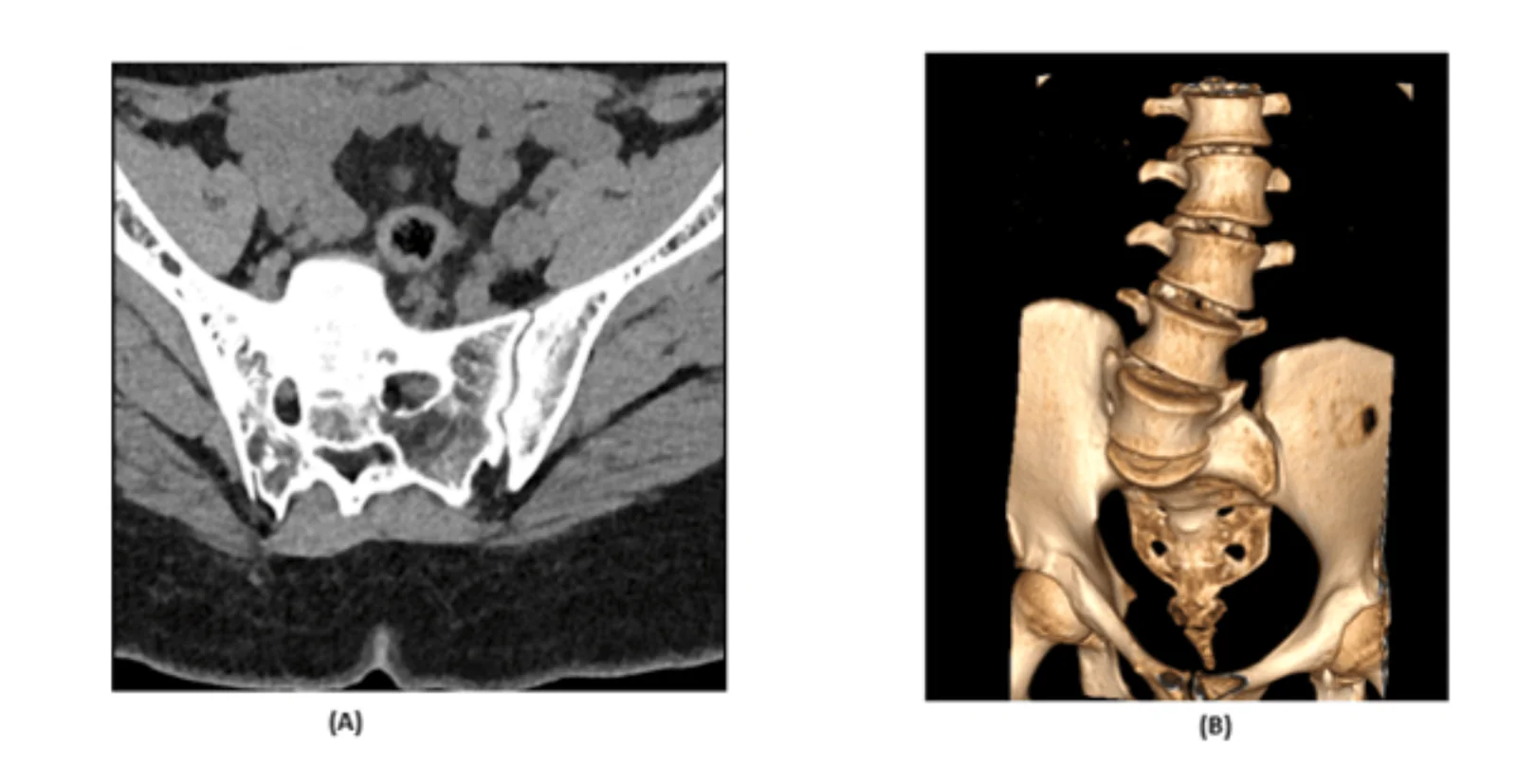
Computed tomography of the sacroiliac joints and the lumbar spine Computed tomography of the sacroiliac joints and the lumbar spine: axial view (A) and 3D reconstruction (B) confirming right hemiagenesis.

Following the clinical and radiological assessment, a neurosurgical consultation was obtained, and no indication for surgical treatment was identified. Conservative management was therefore initiated, including symptomatic treatment with nonsteroidal anti-inflammatory drugs (NSAIDs) and muscle relaxants. The patient also underwent an individualized functional rehabilitation program. The clinical course was favorable; she reported a reduction in pain and an improvement in walking capacity and functional independence.

## Discussion

Sacral agenesis (SA), a major clinical entity within the spectrum of caudal regression syndrome, is a rare congenital anomaly resulting from abnormal development of the caudal mesoderm between the third and fourth weeks of gestation [1].

The reported incidence of sacral agenesis ranges from 1 in 7,500 to 1 in 100,000 live births. A male predominance has also been reported [1-3]. One of the most frequently reported risk factors is maternal diabetes [1]. In our case, no history of maternal diabetes during pregnancy was reported. Other proposed mechanisms include genetic factors, vascular hypoperfusion, and teratogenic exposures [3].

Clinical manifestations vary considerably according to the phenotype and severity of the malformation. Severe forms are often associated with multisystem involvement, particularly neurological and urogenital abnormalities, including neurogenic bladder, fecal incontinence, and motor deficits. In contrast, milder forms may predominantly present with musculoskeletal abnormalities, such as spinopelvic instability, limb deformities, and vertebral anomalies [1,3].

Several classification systems have been proposed for sacral agenesis. The Renshaw classification (1978) remains one of the most widely used and is based on the extent of the residual sacrum and the type of articulation between the spine and pelvis [4]. It distinguishes four types: type I, unilateral partial or complete sacral agenesis; type II, partial, bilateral, and symmetrical sacral agenesis; type III, complete sacral agenesis with variable lumbar agenesis, in which the iliac bones articulate directly with the lowest remaining lumbar vertebra; and type IV, complete sacral agenesis with fusion of the iliac bones beneath the lowest remaining lumbar vertebra. According to the Renshaw classification, our patient’s unilateral partial sacral agenesis corresponds to type I.

Published reports of sacral hemiagenesis diagnosed in adulthood are scarce. Karacalioglu et al. reported a 69-year-old woman with chronic low back pain in whom sacral hemiagenesis was identified on imaging [5]. Morimoto et al. described two younger patients with unilateral partial sacral agenesis associated with ipsilateral developmental hip dysplasia, gait abnormalities, and tethered cord [6]. In contrast, our patient was diagnosed in adulthood and presented predominantly with chronic mechanical lumbopelvic pain and compensatory lumbar scoliosis, without neurological or urogenital abnormalities.

This unilateral sacroiliac abnormality may alter lumbopelvic biomechanics, resulting in asymmetric load distribution across the pelvis [7,8]. Altered sacroiliac mechanics may contribute to chronic low back and buttock pain and compensatory changes in spinopelvic alignment [9]. However, the biomechanical contribution of unilateral sacroiliac hemiagenesis to pain and compensatory lumbar scoliosis in our patient could not be directly assessed, as no specific biomechanical evaluation was performed.

Plain radiography is often the initial imaging examination and may suggest the diagnosis by revealing abnormalities such as sacral arch dysmorphism or pelvic asymmetry. Computed tomography (CT) provides more detailed assessment of the bony anatomy and the extent of the sacral defect, whereas magnetic resonance imaging (MRI) is particularly useful for evaluating associated neural and soft-tissue abnormalities [8,10].

From a therapeutic perspective, the management of mild to moderate forms of sacral agenesis is primarily conservative. Treatment generally relies on a functional approach, including rehabilitation aimed at improving lumbopelvic stability, muscle strengthening, and correction of postural abnormalities. In our patient, conservative management consisted of non-steroidal anti-inflammatory drugs, analgesics, muscle relaxants, and an adapted rehabilitation program. Neurosurgical evaluation found no indication for surgical treatment. Clinical improvement was observed during follow-up; however, quantitative outcome measures were not systematically recorded.

Surgical treatment, in contrast, is uncommon and is generally reserved for severe cases characterized by major spinopelvic instability or associated neurological complications [2,4,8].

The rarity of this unilateral and relatively mildly symptomatic presentation highlights the need for better recognition and characterization of such atypical variants to help reduce diagnostic delay into adulthood.

## Conclusions

Sacral agenesis is a rare congenital malformation characterized by significant clinical variability. This case illustrates an unusual, localized form diagnosed in adulthood after a prolonged history of chronic mechanical low back and buttock pain, without neurological or urogenital involvement. Although rare, congenital sacropelvic abnormalities may be considered in the differential diagnosis of persistent lumbopelvic pain associated with structural asymmetry or unexplained abnormalities in spinal alignment, particularly when the initial evaluation is inconclusive. Imaging plays a key role in establishing the diagnosis and characterizing the anatomical abnormality. In mild to moderate forms without major neurological complications, management is generally conservative and should be individualized according to the patient’s clinical presentation.
